# Combining Mental Training and Physical Training With Goal-Oriented Protocols in Stroke Rehabilitation: A Feasibility Case Study

**DOI:** 10.3389/fnhum.2018.00125

**Published:** 2018-04-03

**Authors:** Xin Zhang, Ahmed M. Elnady, Bubblepreet K. Randhawa, Lara A. Boyd, Carlo Menon

**Affiliations:** ^1^MENRVA Research Group, Simon Fraser University, Vancouver, BC, Canada; ^2^Brain Behaviour Lab, University of British Columbia, Vancouver, BC, Canada

**Keywords:** stroke rehabilitation, mental training, physical training, BCI, exoskeleton, FES

## Abstract

Stroke is one of the leading causes of permanent disability in adults. The literature suggests that rehabilitation is key to early motor recovery. However, conventional therapy is labor and cost intensive. Robotic and functional electrical stimulation (FES) devices can provide a high dose of repetitions and as such may provide an alternative, or an adjunct, to conventional rehabilitation therapy. Brain-computer interfaces (BCI) could augment neuroplasticity by introducing mental training. However, mental training alone is not enough; but combining mental with physical training could boost outcomes. In the current case study, a portable rehabilitative platform and goal-oriented supporting training protocols were introduced and tested with a chronic stroke participant. A novel training method was introduced with the proposed rehabilitative platform. A 37-year old individual with chronic stroke participated in 6-weeks of training (18 sessions in total, 3 sessions a week, and 1 h per session). In this case study, we show that an individual with chronic stroke can tolerate a 6-week training bout with our system and protocol. The participant was actively engaged throughout the training. Changes in the Wolf Motor Function Test (WMFT) suggest that the training positively affected arm motor function (12% improvement in WMFT score).

## Introduction

Stroke is the leading causes of permanent disability in adults in the world (Krebs et al., 1998; Mozaffarian et al., 2016). The literature shows that conventional rehabilitation is able to improve the function of the hemiparetic upper extremity on individuals with chronic stroke (Kwakkel et al., 1997; Hogan et al., 2006; Wing et al., 2008; Krebs et al., 2009). However, rehabilitation can be a long process requiring hard labor with high cost (Mozaffarian et al., 2016). These drawbacks motivated researchers to find solutions to minimize the human labor and thus decrease rehabilitation cost (Freeman et al., 2009; Poli et al., 2013). Most robotic devices are capable of passively delivering a high number of training repetitions to the stroke-affected limb (Freeman et al., 2009; Loureiro et al., 2011; Ren et al., 2013; Herrnstadt et al., 2015; Proietti and Crocher, 2016). However, clinical evidence suggests that users' engagement plays an important role to enable and augment motor recovery (Hogan et al., 2006; Krebs et al., 2009; Lo and Xie, 2012). Therefore, passive repetitive training is insufficient. There is a need for rehabilitation interventions that provide intensive task-specific repetitions with mental engagement to achieve the best possible rehabilitation outcomes.

Brain-computer interface (BCI) can enhance mental engagement in movements via direct communication through brain signals (Wolpaw et al., 2002; Daly and Wolpaw, 2008), forcing concentration on designated tasks (Gentili et al., 2006) Several researchers have shown promising data suggesting that BCI driven robotic devices may be effective for neuro-rehabilitation (Wang et al., 2009; Frisoli et al., 2012; Elnady et al., 2015). However, recent research suggested that combination of mental and physical training would even augment the rehabilitation outcomes (Chaudhary et al., 2016). This research combined a BCI system with other devices that utilized physiological and/or external systems to ensure the learning/re-learning process (Scherer et al., 2007a,b; Pfurtscheller, 2010; Allison et al., 2012; Amiri et al., 2013).

In the current study, we propose a portable stroke rehabilitation platform that combines physical and mental training for stroke rehabilitation. The proposed platform consists of an electroencephalography (EEG) based BCI system for mental training. For physical training, we used a force sensor embedded orthosis for elbow extension/flexion, and a functional electrical stimulation (FES) unit for hand extension. To use this system, the participant has to both imagine the designated task and move the forearm to the designated direction (flexion or extension depends on the context) to trigger the assistance of the orthosis (BCI and force sensor control: BF control for short). The BF control mechanism was designed specifically for combining mental and physical training. We also developed a progressive functional training protocol with three increasing levels of difficulty, to complement with the hardware design. Motor improvements were assessed as clinical outcome measures via Wolf Motor Function Test (WMFT).

## Methods

In order to test the feasibility and possible efficacy of the proposed rehabilitation platform and protocol for stroke rehabilitation, we recruited an individual with chronic stroke. He completed 6 weeks (18 sessions) of training with the proposed platform using BF control. During training, we recorded the success rate of the participant using the platform. The success rate was calculated as the ratio between the successful number of trials in triggering the device assistance and a total number of trials. Further, clinical assessments including WMFT were also recorded every 2 weeks.

### General system setup

**Figure 1:**
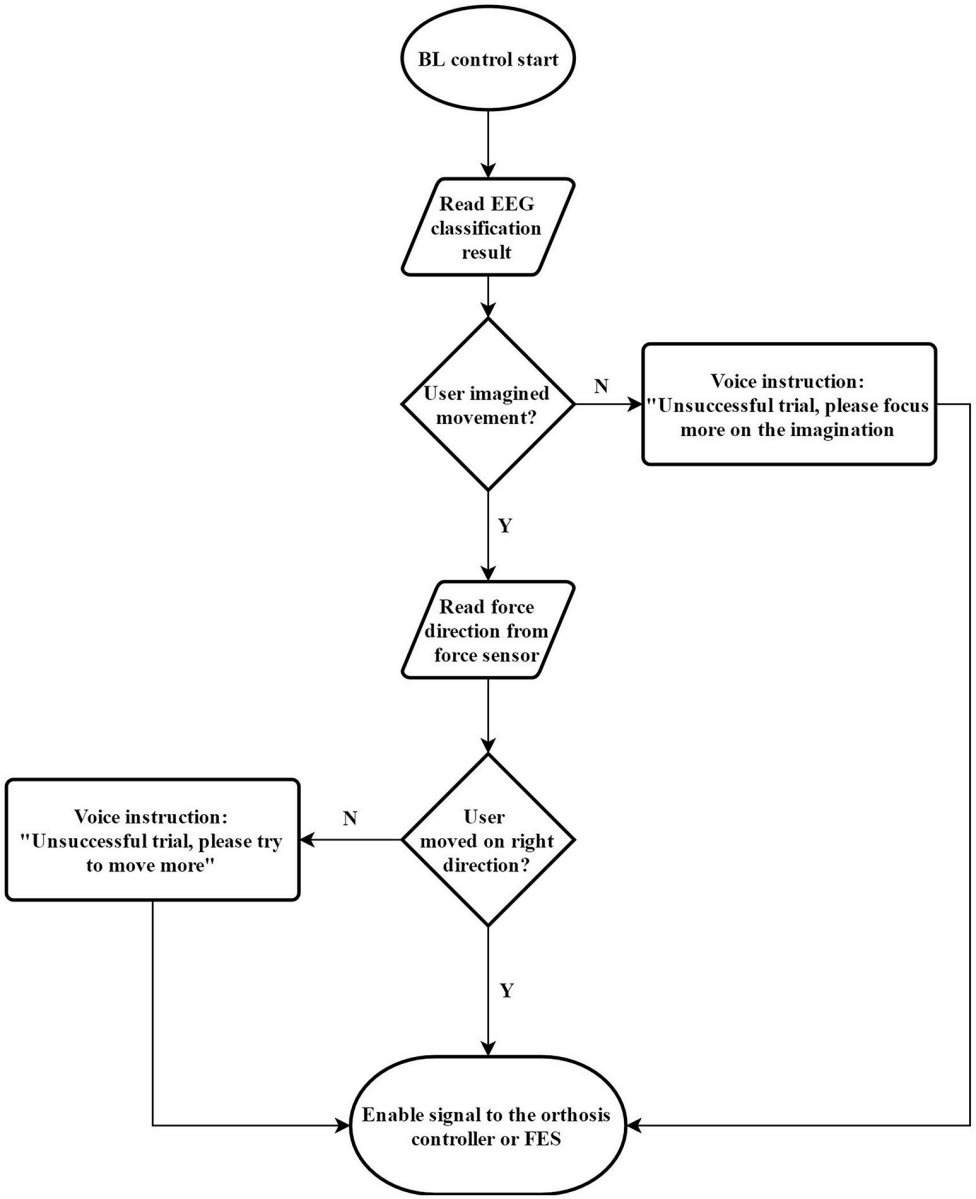
Flow chart for the BF control method, which was used in this paper to combine mental training together with physical training.

We designed the BF control method to ensure the user engagement in both mental and physical training. EEG data were collected to assess mental engagement, while force information was collected to gauge motor output. The BF control flowchart is shown in Figure 1. We used BF control as basic blocks to complete the training tasks in our protocol. However, we have many different options to facilitate our training protocol. For example, we can design our training platform fully via functional electrical stimulation. However, Lew et al. reported that not all individuals with chronic stroke are able to use an FES unit for elbow position control (Lew et al., 2016). Therefore, we did not use a full FES design in this study. We also did not use stationary robotic designs [such as Kinarm (Sanchez et al., 2005) or Harmony (Kim and Deshpande, 2017)], as our objective was to design a portable platform to promote flexibility in rehabilitation. Therefore, we created a unique design consisting of an elbow orthosis to facilitate movement together with an FES unit to activate object-releasing hand movement. The proposed stroke rehabilitation platform was built on top of the BF control method. Each step of the movement in the training was programmed to run the BF control to ensure the participant focus.

#### Elbow orthosis design and development

**Figure 2:**
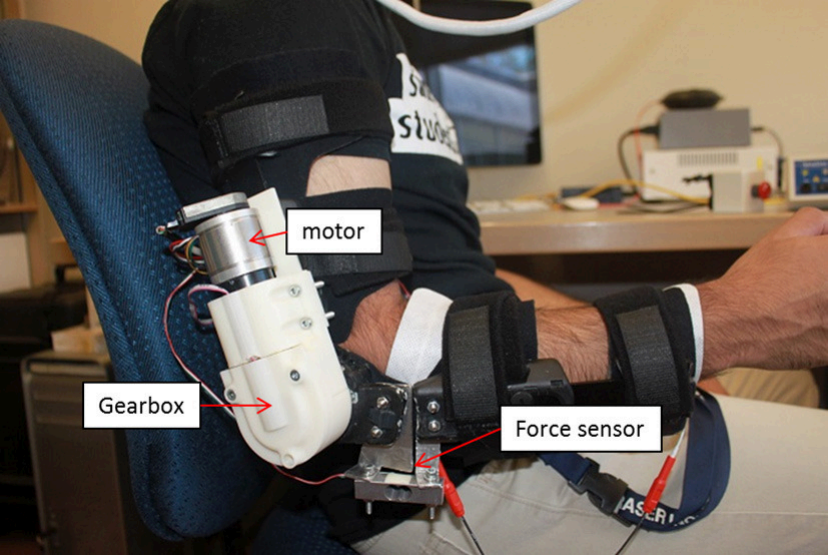
Orthosis used in this study.

The elbow orthosis used in this study is an arm robot prototype developed in our lab (see Figure 2) and modified across multiple versions (Xiao et al., 2014; Elnady et al., 2015). The elbow orthosis was fabricated from an off the shelf brace (Breg T scope Elbow Brace) with mechanical stops and active mechanical components that had one degree of freedom (DOF) for elbow flexion/extension. The orthosis is actuated via a brushless 24-Volts DC (BLDC) motor that provides a torque of 52.7 mN at a nominal speed of 10,200 rpm with 85% max efficiency and two stages of reduction. The first reduction stage was an off the shelf planetary gearhead (From Maxon Motors) has a reduction ratio of 51:1, the second stage was a custom-made single bevel gear set with 3.5:1 reduction ratio.

The angular position of the end effector of the orthosis was measured via a low profile long life EVWAE Panasonic potentiometer. In addition, we used an encoder (HEDL 500 CPT with 3 channels) mounted on the motor side as a redundant sensor for safety purposes. The orthosis was encapsulated in a custom made casing. The casing was rapid-prototyped from acrylonitrile butadiene styrene (ABS) plastic to minimize the weight.

We integrated a micro force sensor (Phidgets 3,133, 0–5 kg) at the end effector of the orthosis to measure the interaction forces between the user and the orthosis. The integrated micro load has a 0.05% precision, 0.05% Non-Linearity of the full scale (FS), 0.05% hysteresis of FS, and 0.1% Creep of FS (per 30 min). These features enable the accurate measurement of the human and the orthosis interaction forces. Theoretically, the orthosis is capable of providing a total torque of 9.4 Nm at a nominal speed of 57 rpm, with a total weight of 930 gm, and a total range of motion between 0 and 130°. However, for safety purposes, we limited the range of motion to only 30–120° via the mechanical stops of the brace.

There are several advantages of the proposed orthosis. First, the orthosis is lightweight (≈900 grams) and portable. Second, the user can don or doff the orthosis in <30 s when aided. Lastly, the orthosis's joint does not to interfere with the user's natural arm position when he/she is relaxed or performing tasks. These features are desirable and enhance the usability of the orthosis when assisting users in performing goal-directed movements in daily activities and exercises for rehabilitation purposes.

#### Functional electrical stimulation

We used a functional electric stimulation (FES) unit (RehaStim, Hasomed Inc., Germany) to assist wrist/hand extension. The FES unit generates symmetrical biphasic pulses, with a fixed frequency of 35 Hz and peak duration of 150 μs. The participant was required to wear two self-adhesive electrodes on the forearm of the impaired arm to aid wrist and finger flexion (grasp action). The stimulus amplitude of the FES was incrementally tested with the participant until the impaired hand is fully and comfortably extended.

#### EEG acquisition and classification

We used a 32-channel, EGI Geodesic N400 system (Electrical Geodesics Inc., USA) to record the EEG data from the participant. EEG acquisition and analysis was a two-step process: (1) collect EEG data to obtain a BCI model of the participant; and (2) utilize the obtained model to classify the participant's intentions in real time.

For a model generation, we used ‘Stimulus Presentation’ mode in BCI2000 (Schalk et al., 2004), where the EEG was recorded and filtered with a bandpass filter of 1–45 Hz at 1 kHz. In this stimulus presentation mode, two different visual cues were displayed on the computer monitor. The first cue was a cross-sign in the middle of a white screen. During the cross picture on the screen, the participant was asked to keep his eye on the cross and relax. The second cue was a picture of an elbow; the participant was asked to perform the kinesthetic motor imagery for elbow extension and flexion for at least two repetitions. Kinesthetic motor imagery means that the participant was asked to imagine himself performing the movement and focusing on the sensation of the movement (Neuper et al., 2005). Each Stimulus Presentation run consisted of 20 randomized cues (rest or elbow). Each cue was shown on the monitor for 4 s, followed by randomized intervals of 4–6 s (relax intervals). The participant was required to complete five runs of stimulus presentation. We managed to collect 50 trials of the participant's EEG data during motor imagery of elbow movement and 50 trials of rest.

In order to obtain the BCI control model, the data were analyzed offline using BCILAB (Kothe and Makeig, 2013), a BCI toolbox based on Matlab (The MathWorks, Inc., USA). First, data were resampled at 250 Hz. Then, a finite impulse response (FIR) band-pass filter was used to filter out the 6–35 Hz frequency band. This frequency band covers the mu and beta rhythms, which is reported to contain ERS and ERD activities during motor imagery (Pfurtscheller and Lopes da Silva, 1999).

We exploited a searching method to search the EEG data from 0.5 to 3 s after each visual cue, with 2 s of window size and 0.5 s of step size. For each EEG epoch, Band Power (BP) (Pfurtscheller and Neuper, 2001), Common Spatial Pattern (CSP) (Ramoser et al., 2000) and Filter Bank Common Spatial Pattern (FBCSP) (Kai Keng Ang et al., 2008) were independently used as feature extraction, and then a grid search for the best combination of the feature algorithm and classifiers was performed. In this study, Linear Discriminant Analysis (LDA), Dual Augmented Lagrangian (DAL) method and support vector machine (SVM) were used as potential searched classifiers. Detailed feature settings are shown in Table 1. Hyper-parameters of the classifiers also included in the grid search, with a range from 2^−15^ to 2^10^ and the step size was 2 times. The three feature extraction algorithms and three classifiers were tested with all possible 9 feature-classifier combinations with a 10 × 10 cross-validation.

**Table 1:** Feature settings during model training.

**Feature algorithm**	**BP**	**CSP**	**FBCSP**
Frequency Band	**6–32 Hz**	**6–32 Hz**	**6–1 5Hz; 15–25 Hz; 25–32 Hz**
Feature Dimension	**17**	**6**	**18**

During the offline data classification, 54 binary models were generated. The model with the highest cross-validation accuracy was saved for later use. During the online classification, the EEG signal was filtered with the same FIR 1–45 Hz bandpass filter. Then the signal was streamed to a buffer and the pre-acquired model was applied on the buffered EEG signal.

Decisions were obtained once every 500 ms with a moving average among the latest 8 decisions. If the average value was greater than the preset activity threshold, enable command would be sent to the orthosis control module. The activity threshold may vary among different sessions, due to the contacts of our EEG acquisition station was using saline solution. In order to get the proper activity threshold and to minimize false positive, the participant was asked to complete EEG data collection and analysis before the actual training. We asked the participant to rest without closing their eyes for 30 s, while the EEG data was collected and the online classification completed (output every 500 ms). The activity threshold was set as 0.1, higher than the max output value from the classifier in the online classification. Through this process, the possibility of artifact contamination in the BCI control was minimized.

### Inclusion criteria

Our inclusion criteria included: 1) age range from 35 to 85 years, (2) post-stroke duration ≥6 months, (3) MoCA ≥25 (Aggarwal, 2010) (4) shoulder active range of motion (ROM) in all directions of 10–15°, (5) elbow passive extension and flexion ROM of 0–130°, (6) wrist passive extension ROM of 0–15°, and (7) passive full extension for fingers. We searched for a potential participant in our stroke database. Potential participants were excluded if they had; (1) other neurological conditions in addition to stroke, (2) unstable cardiovascular disease, or (3) other serious diseases that precluded them from undergoing the study (i.e., undergoing other studies etc.,). Next, we contacted participants to determine if they could commit to a 6-week intensive training protocol. Finally, we chose one participant, a 37-year-old male with aphasia who was 11 years post-stroke.

### Assessment tests

For pre-assessment, we used three baseline assessments (BLA), each performed 2 weeks apart. During the training session, participant went through a battery of tests again every 2 weeks. The primary outcome measure was WM assessment. Other secondary outcome measures were: Fugl Meyer Assessment (FMA) and the success rate of triggering the device during each training day. The participant was required to complete the WMFT and FMA every other week as clinical outcome assessments by a “blind” test administrator, who was neither aware of, nor involved in, the study protocol.

### Brain symmetry index (BSI) of the participant

In addition to the WMFT, we were also interested in understanding if the training outcome could be reflected in EEG. The BSI was introduced to assist the visual interpretation of the EEG, in particular, to quantify both the spatial (left-right) and the temporal spectral characteristics. Previously the BSI has been applied in monitoring during carotid endarterectomy, acute stroke and focal seizure detection (van Putten, 2007). Other work showed that BSI is negatively correlated with participant's functional motor outcomes (i.e., the higher the BSI, the lower the FMA) (van Putten and Tavy, 2004; van Putten et al., 2004; van Putten, 2007; Anastasi et al., 2017).

(1) $$\begin{array}{c} BSI(t)=\;\frac{1}{k}\sum_{n=1}^{k}|\frac{R_{n}^{*}(t)-L_{n}^{*}(t)}{R_{n}^{*}(t)+L_{n}^{*}(t)}| \end{array}$$

Where k is the number of discrete frequencies, and

(2) $$R_{n}^{*}(t)=\frac{1}{m}\;\overset{m}{\underset{ch=1}{\sum^{\;}}}a_{n}^{2}(ch,t)$$

$R_{n}^{*}(t)$ is for the channels on the right hemisphere. A similar equation was used for the channels on left hemisphere [$L_{n}^{*}(t)$]. In this equation, $a_{n}^{2}\left(ch,t\right)$ is the Fourier coefficient with index n of channel ch, at time t, corresponding to a particular event epoch [t-T, t]. In this paper BSI was calculated with *T* = 4 s, both at rest and during motor imagery.

### Training protocol

The total training duration was 6 weeks. Each week consisted of 3 sessions of training sessions (approximately one and a half hour) on alternate days. The full study schedule is shown in Table 2.

**Table 2:** Training and testing schedule for the case study.

**Training schedule**	**Baseline assessment**			**Training weeks**						**Retention**
	**D1**	**D1+2W**	**D1+4W**	**TW1**	**TW2**	**TW3**	**TW4**	**TW5**	**TW6**	**TW6 + 4W**
Assessments (WMFT, FM).	√	√	√	√	√		√		√	√
Stimulus presentation	√			√						
Training protocol				Warm-up	Level 1	Level 1	Level 2	Level 2	Level 3	

*TW, training week; D, day; W, week*.

#### Warm-up training (training week 1)

During the warm-up training (Training week 1), three basic sessions (described below) were introduced to the participant. The aim of this warm-up training was to familiarize the participant with the orthosis system and the basic BCI control methods.

In warm-up training session 1, no engagement was required from the participant. This session involved passive movements of the elbow flexion-extension (using orthosis) and hand opening (using FES). The training lasted for 30 min for each movement (elbow and hand). Each movement was repeated 25 times. Session 1 was designed to familiarize the participant with the orthosis and ensure the participant's range of motion on the hemiparetic upper limb could tolerate the range of the orthosis.

In a warm-up training session 2, the participant was required to trigger the orthosis using only kinesthetic motor imagery. This session involved active movements of the elbow flexion-extension (using orthosis) and hand opening (using FES) controlled by the participant through EEG. If the participant was unable to trigger the device within the designated time, the device would passively move the participant's arm to receive minimal training. The training lasted for half an hour for each movement (elbow and hand). The minimum number of repetitions for each movement was 10 times if all trials were unsuccessful. Session 2 was designed to familiarize the participant with BCI control and obtain the activity threshold for the EEG online classification.

In a warm-up training session 3, the participant repeated the same movements as in session 2 using different control mechanism. For elbow movement, the participant was required to concentrate on imaging opening/closing elbow and then move his elbow toward the designated direction (BF control). For hand and wrist control, the participant was required to concentrate on imaging opening the hand to switch on the FES that assists in opening the hand, the FES was designed to switch off automatically after 5 s. Session 3 was designed to get the participant familiarized with the basic control components of the goal-oriented protocols proposed in the training sessions.

#### Goal-oriented training tasks (training weeks 2–6)

The training from the second to the sixth week required the participant to complete 12 days in which four different goal-oriented tasks were practiced. Each task was assisted by the orthosis, which could be triggered by the BF control. The functional tasks were split into three levels of difficulty. Level 1 included only elbow movement, simple flexion/extension. Level 2 included: a task using both hands to improve bilateral control and coordination. Level 3 included: reach, grasp, place, and release an object.

**Figure 3:**
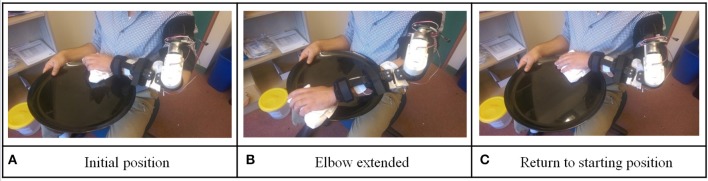
Illustration for level 1 training protocol: plate cleaning task.

Level 1 task, plate-cleaning task: the participant was requested to wear the orthosis and hold the plate in a horizontal position close to the trunk with the non-paretic arm (as shown in Figure 3A). Then the participant was required to place the paretic arm proximal to the trunk and above the plate. This was defined as the initial position. At the end of each training repetition, the device would return to this position. Vocal instructions from the device would instruct the participant to imagine the sensation of moving elbow to wash the plate and physically extend his elbow (to meet the criteria for BF control). If the participant successfully passed the BF control check, the orthosis would assist the participant to perform elbow extension (as shown in Figure 3B). If the participant failed to pass the BF control check within 10 s after vocal instructions, the device would automatically extend the participant's elbow and inform the participant this was an unsuccessful trial. After extending the participant's elbow, the device would ask the participant to flex his elbow to complete the task cycle (as shown in Figure 3C). Same BF control checking method was used to ensure the participant was engaged in the training.

**Figure 4:**
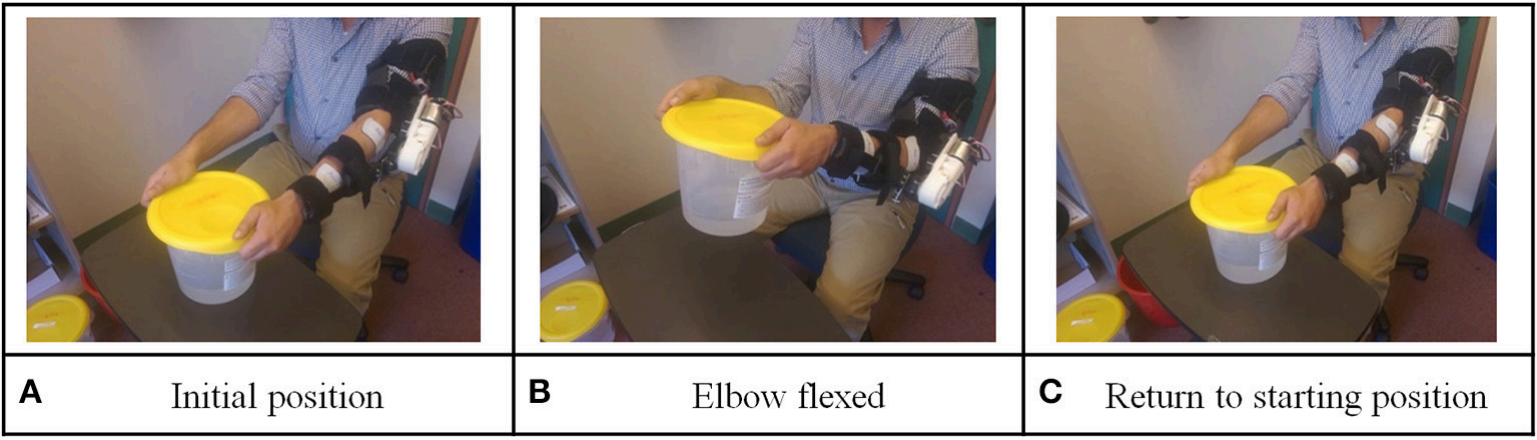
Illustration for level 2 training protocol: lifting and placing task.

Level 2 involved lifting a bucket: the participant was requested to wear the orthosis, extend both of his arms and hold a bucket (as shown in Figure 4A). This was defined as the initial position. At the end of each training repetition, the device returned to this position. Again as in level 1, vocal instructions from the device instructed the participant to imagine the sensation of flex elbow to lift the bucket and physically flex his elbow. If the participant successfully passed the BF control check, the orthosis would assist the participant to perform elbow flexion to lift the bucket (as shown in Figure 4B). If the participant failed to pass the BF control check within 10 s after the vocal instructions, the device would automatically flex the participant's elbow and inform the participant this was an unsuccessful trial. After flexing the participant's elbow, the device would ask the participant to extend his elbow to put the bucket on the desk (as shown in figure 4C). Same BF control checking method was used to ensure the participant was engaged in the training.

**Figure 5:**
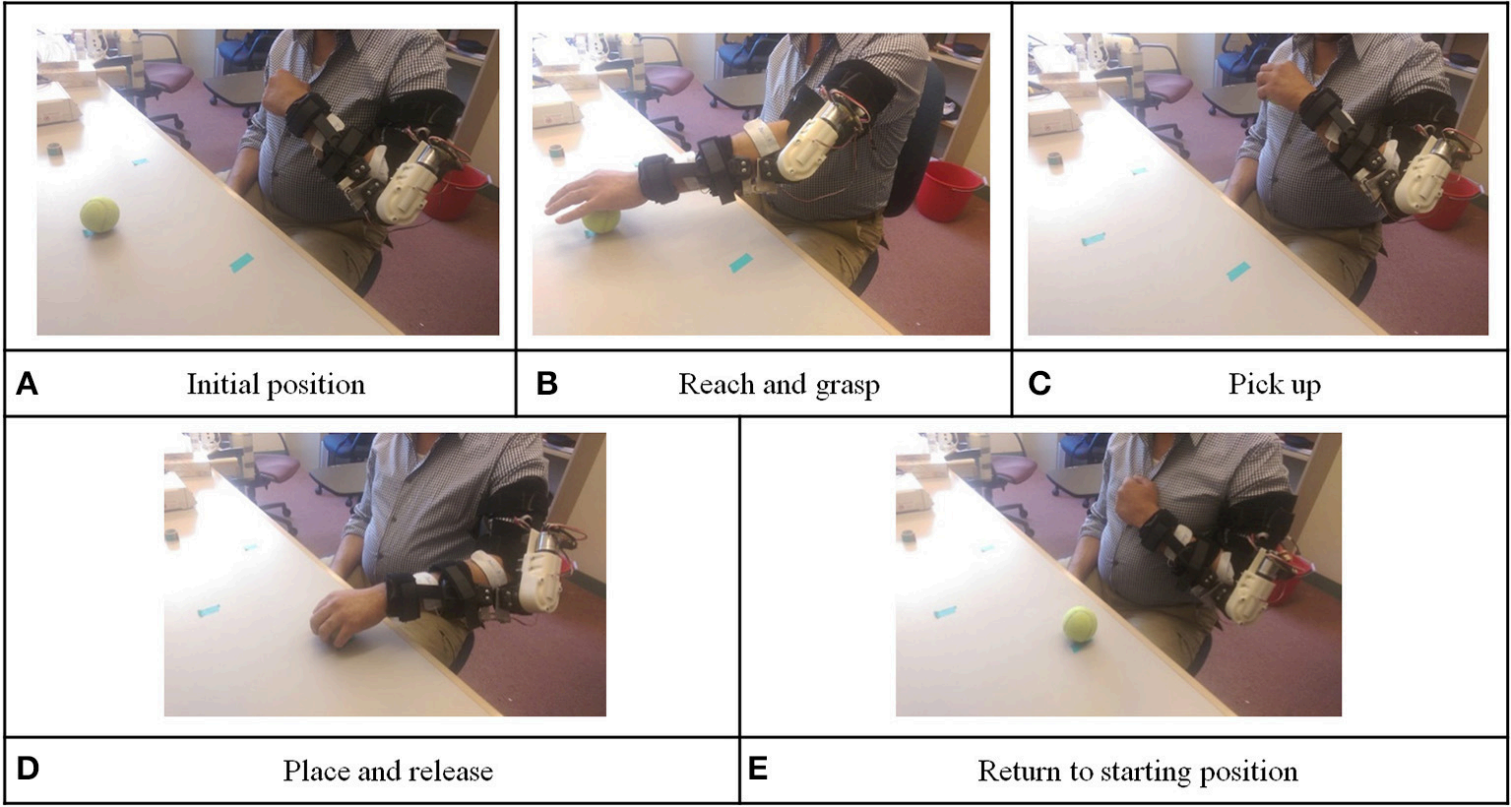
Illustration for level 3 training protocol: picking-up and placing task.

Level 3 involved a placing and releasing task: In this level, we added FES unit to assist with hand control. The participant wore the orthosis and FES electrode and held his paretic hand in front of his chest. This was defined as the initial position (as shown in Figure 5A). At the end of each training repetition, the device returned to this position. As in level 1 and 2 vocal instructions from the device instructed the participant to imagine the sensation of extending his elbow to reach and grab the target object and physically extend his elbow and open the hand. The FES unit would assist to open the participant's hand. The device would wait for 3 s, and the FES unit would be switched off so that the participant could hold the object (as shown in Figure 5B). If the participant failed to pass the BF control check within 10 s after the vocal instructions, the device would automatically extend the participant's elbow, open the hand, and inform the participant this was an unsuccessful trial. After grasping the object, the device would give vocal instruction to ask the participant to flex his elbow to pick up the ball from the desk (as shown in Figure 5C). The same BF control checking method was used to ensure the participant was thinking about the elbow movement and moving toward the correct direction. Then, the device would give vocal instructions to imagine elbow extension and physically extend his elbow to place the object down. If the participant successfully passed the BF control check, the orthosis would assist the participant to perform elbow extension. After the orthosis reached the designated extension angle, the FES unit switched on, so that the participant could release the object in his hand (as shown in Figure 5D). Again, the device asked the participant to imagine elbow flexion and physically flex his elbow to move his hand back to the initial position. BF control checking was also used in this phase (as shown in Figure 5E).

During training, a trial was considered successful only if the participant was able to trigger both EEG system with motor imagery and bend the force sensor in the correct direction of the required movement of the elbow. If the participant for any reason did not have a successful trial, the orthosis system would perform the required movement by moving the participant's limb passively to maintain a minimum number of delivered repetitions (10 repetitions).

### Statistical analysis

We statistically analyzed six sessions of the WMFT score collected during this study. First, we fit the WMFT scores on a natural logarithm regression model according to the least square method, to assess the trend associated with our training protocol. Next, a linear hypothesis tested the statistical significance of the regression. Since the WMFT has high inter-rater and test-retest reliability (Morris et al., 2001), we assumed that the error between WMFT data we collected and the regression model was normally distributed.

We also calculated BSI from the participant's EEG data, and correlations between the WMFT and BSI during both rest and motor imagery states were investigated. The correlations indicate whether our claims on the changes in the WMFT scores were actually reflected on the EEG level. Both Pearson's correlation and Spearman's correlation were calculated between WMFT and BSI.

## Results

### BCI performance

**Figure 6:**
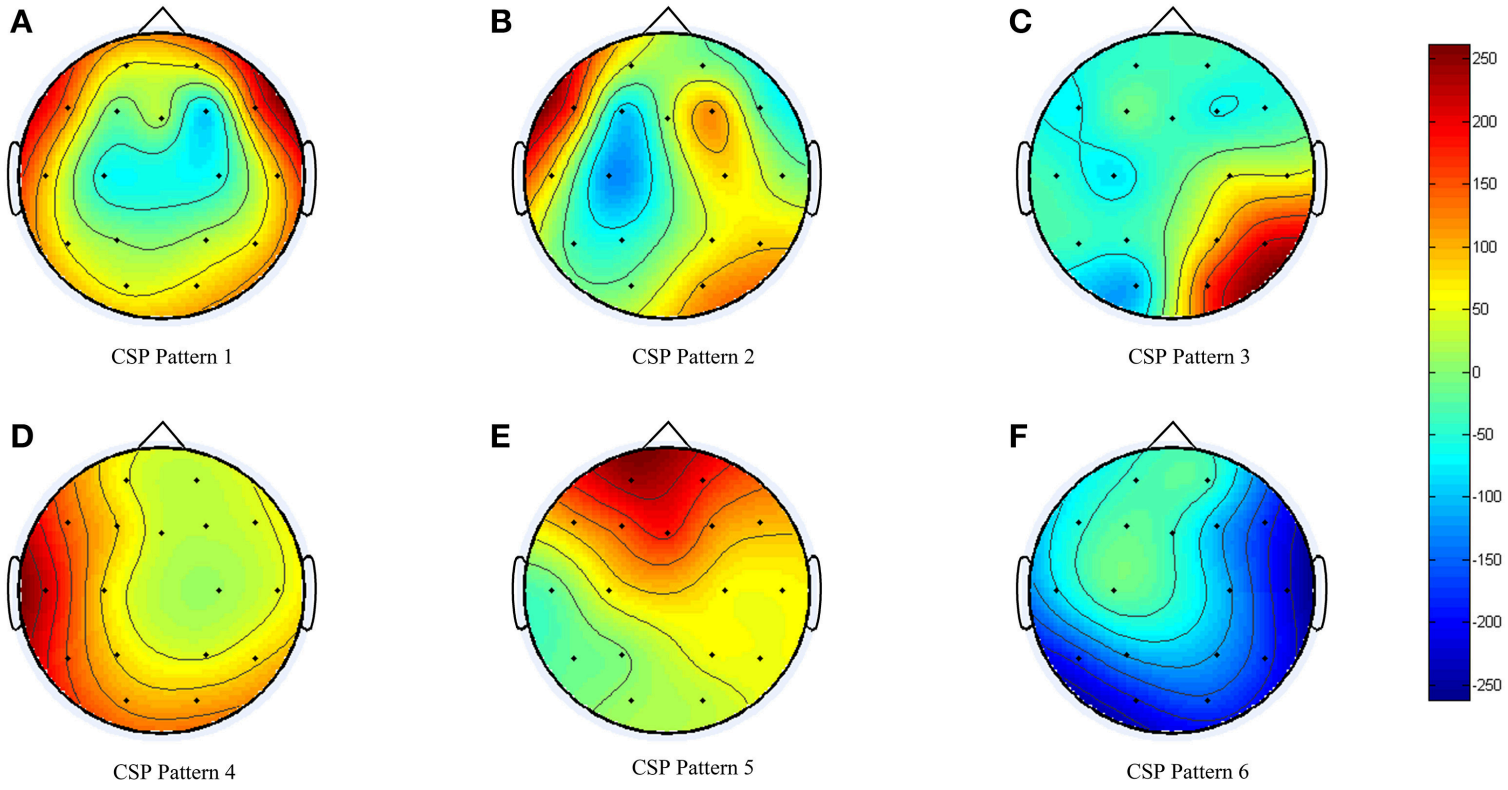
CSP model obtained for the participant.

During the BCI model training (obtaining or generation), the EEG data collected was sent to three types of feature extraction algorithm and cross-validated with three types of classifiers. For the participant in this study, the CSP feature algorithm together with LDA classifier returned the highest cross-validation accuracy of 80.1%. The spatial filter obtained is shown in Figure 6. Clear event-related desynchronization (ERD) was captured by the machine-learning algorithm in Figure 6B.

### Success rate

According to the training protocol, the device can either facilitate active training (BCI or BF control) or passive training. The success rate was introduced to measure the control accuracy during the training. The success rate was calculated as the ratio of successfully controlled trials, by the participant, in the total training trials within one training day. For example, if the participant was missing one control clue during one trial, this trial was not counted as successful. The calculated success rate on each training day was averaged and summarized for each training week (Table 3).

**Table 3:** Success rate in triggering the system.

	**BCI success rate**	**BL control success rate**
Week1	0.684 ± 0.048	0.410
Week2	0.771 ± 0.138	0.497 ± 0.152
Week3	0.653 ± 0.215	0.453 ± 0.076
Week4	0.74 ± 0.124	0.697 ± 0.095
Week5	0.936 ± 0.022	0.826 ± 0.017
Week6	0.906 ± 0.111	0.831 ± 0.133

The participant was a BCI novice. In the first training week, the participant's success rate was 68.4% with BCI control, and his success rate for BF control was only 41.0%. However, after 6 weeks of training, the participant managed to achieve a success rate of 90.6% for BCI control and 83.1% for BF control.

### WMFT and FMA result

According to the inclusion criteria, the participant had shoulder active range of motion in all directions of 10–15°. The participant was required to complete the WMFT and FMA every other week by a “blind” test administrator, who was neither aware of, nor involved in, the study protocol. The first three sets of WMFT data were collected as baseline measurements without training involved.

WMFT scores are shown in Table 4. Data were omitted if the participant was not able to finish the task throughout the baseline measurement and the 6 weeks of training. The baseline assessment showed that the participant was not able to finish Extend-elbow (side), Extend-elbow (weight) and Hand-to-box (front). The participant was also not able to several fine motor movements including Lift-can, Lift-pencil, Lift-paper-clip, and Stack-checkers

**Table 4:** Wolf Motor scores of the participant.

**No**.	Assessment content^*^	**Unit**	**Right hand assessment**							
			**Baseline**			**Assessment in training weeks**				**Retention**
			**1**	**2**	**3**	**1**	**2**	**3**	**4**	**5**
1	Forearm to table (side)	seconds	3.62	3.83	3.72	3.44	3.08	4.24	3.74	4.13
2	Forearm to box (side)	seconds	120.00	120.00	16.91	120.00	16.02	120.00	14.88	9.22
5	Hand to table (front)	seconds	2.17	5.01	3.49	2.23	3.205	4.55	4.69	2.58
6	Hand to box (front)	seconds	120.00	120.00	120.00	8.57	10.28	27.36	30.51	4.78
7	Weight to box (highest)	lbs	0.00	0.00	0.00	3	2	2	2	3
8	Reach and retrieve	seconds	6.36	4.17	3.26	7.04	2.80	2.98	4.13	16.81
14	Grip strength (mean)	kg	7.12	4.34	9.29	6.34	9.47	4.43	6.45	6.37
16	Fold Towel	seconds	120	82.56	120	120.00	89.22	71.81	120.00	120
17	Lift Basket	seconds	4.16	9.52	7.82	9.45	5.74	6.06	8.34	7.49

* The tasks which participant was not able to finish throughout the study, were not included in this table

WMFT results show that participant was still not able to complete all the tasks, after the training. Therefore, he scored 120 s (max allowed time) for hand to box in the baseline and first practice sessions. Major improvements were observed for Forearm-to-Box (side) by 89%, Hand-to-Box (front) by 96% and Weight-to-Box. The participant also showed minor improvements in Hand-to-table (28%), when retention score was compared to the baseline data (third session). The participant's score showed major fluctuations on Forearm-to-Box task and Fold-Towel task.

The detailed FMA score is shown in Table 5. Only minor fluctuations were observed during and after the training of this study.

**Table 5:** Fugl Meyer Assessment score of the participant.

	**Baseline assessment**			**Assessment1**	**Assessment2**	**Assessment3**	**Assessment4**	**Retention**
Right Arm	22	23	22	19	19	22	21	22
Left Arm	62	64	64	64	62	62	62	62

### Statistical analysis

#### WMFT score regression analysis

**Figure 7:**
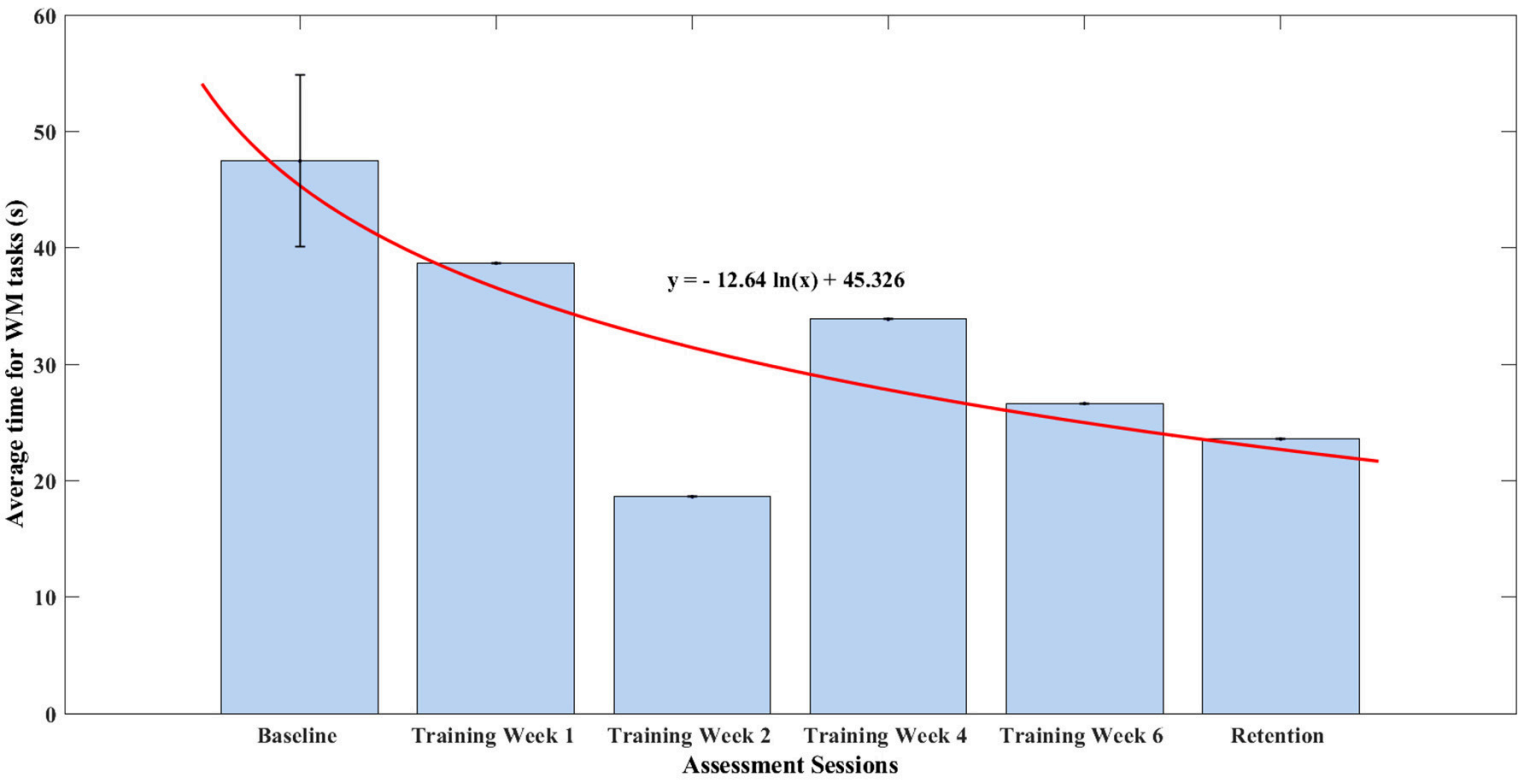
Summary for average time to finish WM tasks.

In this section, all the items measuring time in the WMFT were taken and an average time of finishing one task of the WMFT was calculated. The baseline assessment session consisted of three assessments, thus, a standard deviation was shown in Figure 7. The remaining assessments were only one-time assessments. The average time in finishing each task of WMFT was summarized in Figure 7. After training, the participant managed to decrease his average time in finishing all items of the WMFT test by 12%, which was 11.17 s. The average time for WMFT tasks fit well into a monotonic decreasing natural logarithm function (*r* = 0.789). According to linear hypothesis test, the result was statistically different (*p* = 0.0103).

#### Correlation between BSI and WMFT score

**Figure 8:**
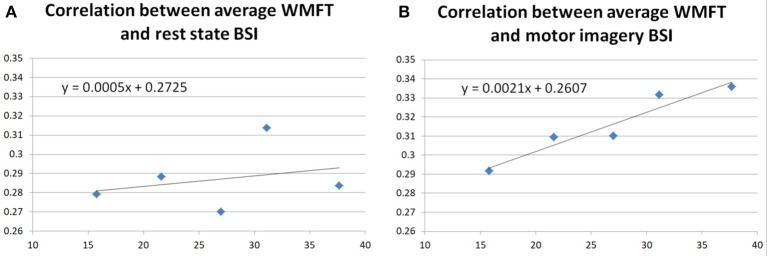
Correlation between the participant's average WMFT score and BSI.

In this paper, the participant's BSIs were calculated both in the rest state of the participant and motor imagery state according to the equations in section Brain Symmetry Index (BSI) of the Participant. Correlations between the WMFT and BSI during both rest and motor imagery states were investigated, to further assess the improvement in the WMFT data. The regression results are shown in Figure 8.

Figure 8A shows the regression correlation between averaged WMFT score and resting state BSI. Both Pearson's correlation (*r* = 0.2790, *p* = 0.6494) and Spearman's correlation (*r* = 0.3000, *p* = 0.6833) indicated very low correlation between the two. Figure 8B shows the correlation between averaged WMFT score and motor imagery state BSI. Both Pearson's correlation (*r* = 0.9568, *p* = 0.0107) and Spearman's correlation (*r* = 1.0000, *p* = 0.0167) indicated very high correlation.

## Discussion

The current review focused on the feasibility of the proposed BCI training platform over 6 weeks of progressive training. The participant's left hemisphere was affected by stroke. Therefore, in the stimulus presentation session, the participant was required to imagine movements of his right upper-limb. Based on the spatial patterns obtained from the offline analysis (Figure 6), we can see that the BCI system was able to capture the ERD for motor imagery (Figure 6B). Considering the participant had no prior experience with BCI, the offline analysis accuracy (80.1%) was reasonable. The participant was actually able to control the device with motor imagery. During the training process, we switched to a higher level of training protocols in Week 3 and Week 5. We also noted variability in the success rate decrease in some training weeks; for example, there was 11.8% decrease in BCI, 4.4% decrease in BF control for Week 3, and 3.0% decrease in BCI for Week 6. The success rate decrease was consistent with the training protocol changes, and the participant managed to quickly adapt to new challenges. The results strongly suggest that both device and protocol were well tolerated by the participant and that training with our device is feasible.

Further, we successfully tested the device over 6 weeks of training. In the baseline assessments, the participant showed very limited ability to functionally use his arm as measured by the WMFT tasks. For example, the participant was not able to complete Hand-to-box (front) and Lift-can tasks. Over the intervention time frame, the participant showed major improvements in the primary outcome measure. The WMFT quantifies upper extremity motor function through timed movement tasks. In this study, the participant was able to improve both timing and strength in selected tasks. He improved most on the Hand-to-Box task and Weight-to-Box task on the stroke affected side, which suggested he had improved his control on the shoulder, elbow, and wrist joints of the affected side. Those improvements were clinically meaningful according to Lin et al. (2009). The participant showed minor improvements in other tasks that are strength based including Forearm-to-Box, Hand-to-Table, and Lift-Basket. However, there was no sign of improvement on fine motor tasks of his affected side. There could be several explanations for the low improvements on the impaired hand. One could be our BF control mechanism was mainly designed to work on the elbow joint, therefore, the participant inherently gained more training of this joint. Another explanation could be distal digit functions are hard to rehabilitate, or that the participant needed a higher dose of the training. However, considering the participant was spending about 1 h in each training session, and the participant was reporting fatigue both mentally and physically after each session, extending the length of the sessions may not be applicable. The fluctuations in the performance of other WMFT tasks for this chronic stroke survivor also suggests the participant was on “the margin” of completing those tasks within the required time, perhaps he could have continued to improve with more training. Finally, it is possible that the neural substrates that support fine motor movement (i.e., the corticospinal tract) as severely damaged by the stroke and not capable of supporting any recovery.

Additionally, BSIs were calculated from the participant's EEG signal, both for the rest state and the motor imagery state. In the literature, BSI was negatively correlated with the functional outcomes (Fugl Meyer score) of the stroke survivors (Anastasi et al., 2017). In this study, no correlation was found between the rest state BSI and averaged WMFT. This might be related to the participant's relatively stable performance on the FMA score, shown in Table 5. In Figure 8, a strong positive correlation was found between motor imagery state BSI and averaged WMFT score (*r* = 0.9568, *p* = 0.0107). In theory, a low BSI score suggests less symmetry on the EEG of two hemispheres. Although motor imagery in healthy should cause unbalanced activity between two hemispheres. However, other studies have suggested motor recovery comes with increased ipsilateral hemisphere movements (Chollet et al., 1991; Weiller et al., 1992; Caramia et al., 1996; Cramer et al., 1997; Honda et al., 1997; Cao et al., 1998; Cuadrado et al., 1999; Morris et al., 2001; Belardinelli et al., 2017). Therefore, the positive correlation between motor imagery BSI could provide evidence that the participant had actual improvement during the training.

Based on these results, we contend that the participant gained control, coordination, and strength in some of the shoulder, elbow, and forearm joint/muscles with repetitive goal-oriented training over a 6-week period.

The population limitation of this study is the primary limitation of this study. Since this is a case study, our results cannot be generalized. Although we successfully tested the feasibility of our device and protocol, the efficacy needs to be further evaluated with a larger population with varied stroke severity and at different times post-stroke in future studies. Our ability to statistically analyze our outcomes is limited by our case study design. Finally, our training platform cannot be independently set-up by the user, and time to set up is half an hour. This problem may limit the proposed platform's application.

## Conclusion

This paper presents a novel rehabilitation platform combining mental and physical training for post-stroke rehabilitation. The proposed training platform together with its assorted training protocol was well tolerated by an individual with chronic stroke during 6-weeks of training. By the end of the training, the participant was able to utilize EEG and force sensor to control the orthosis to finish the training tasks at a very high success rate (90.6% for the BCI control, 83.1% for the BF control). The participant improved his motor function after the training with reduced overall WMFT time. The preliminary results of this case study suggest combining mental and physical training is feasible for patients with chronic stroke.

## Ethics statement

All the methods within this study were in compliance with the Declaration of Helsinki. The study was also approved by the Simon Fraser University (SFU) Office of Research Ethics. Ethic No. 2012s0711.

## Author contributions

Study conception and design: CM, XZ, and AE; Acquisition of data: XZ; Analysis and interpretation of data: XZ; Drafting of manuscript: XZ, AE, and BR; Critical revision: CM and LB.

### Conflict of interest statement

The authors declare that the research was conducted in the absence of any commercial or financial relationships that could be construed as a potential conflict of interest.
